# Disrupted Functional Connectivity of the Amygdala Predicts the Efficacy of Non-steroidal Anti-inflammatory Drugs in Migraineurs Without Aura

**DOI:** 10.3389/fnmol.2022.819507

**Published:** 2022-02-24

**Authors:** Heng-Le Wei, Chen-Hui Xu, Jin-Jin Wang, Gang-Ping Zhou, Xi Guo, Yu-Chen Chen, Yu-Sheng Yu, Zhen-Zhen He, Xindao Yin, Junrong Li, Hong Zhang

**Affiliations:** ^1^Department of Radiology, The Affiliated Jiangning Hospital of Nanjing Medical University, Nanjing, China; ^2^Department of Neurology, The Affiliated Jiangning Hospital of Nanjing Medical University, Nanjing, China; ^3^Department of Radiology, Nanjing First Hospital, Nanjing Medical University, Nanjing, China

**Keywords:** migraine, amygdala, functional connectivity, machine learning, non-steroidal anti-inflammatory drugs

## Abstract

Machine learning (ML) has been largely applied for predicting migraine classification. However, the prediction of efficacy of non-steroidal anti-inflammatory drugs (NSAIDs) in migraine is still in the early stages. This study aims to evaluate whether the combination of machine learning and amygdala-related functional features could help predict the efficacy of NSAIDs in patients with migraine without aura (MwoA). A total of 70 MwoA patients were enrolled for the study, including patients with an effective response to NSAIDs (M-eNSAIDs, *n* = 35) and MwoA patients with ineffective response to NSAIDs (M-ieNSAIDs, *n* = 35). Furthermore, 33 healthy controls (HCs) were matched for age, sex, and education level. The study participants were subjected to resting-state functional magnetic resonance imaging (fMRI) scanning. Disrupted functional connectivity (FC) patterns from amygdala-based FC analysis and clinical characteristics were considered features that could promote classification through multivariable logistic regression (MLR) and support vector machine (SVM) for predicting the efficacy of NSAIDs. Further, receiver operating characteristic (ROC) curves were drawn to evaluate the predictive ability of the models. The M-eNSAIDs group exhibited enhanced FC with ipsilateral calcarine sulcus (CAL), superior parietal gyrus (SPG), paracentral lobule (PCL), and contralateral superior frontal gyrus (SFG) in the left amygdala. However, the M-eNSAIDs group showed decreased FC with ipsilateral caudate nucleus (CAU), compared to the M-ieNSAIDs group. Moreover, the M-eNSAIDs group showed higher FC with left pre-central gyrus (PreCG) and post-central gyrus (PoCG) compared to HCs. In contrast, the M-ieNSAIDs group showed lower FC with the left anterior cingulate cortex (ACC) and right SFG. Furthermore, the MwoA patients showed increased FC with the left middle frontal gyrus (MFG) in the right amygdala compared to HCs. The disrupted left amygdala-related FC patterns exhibited significant correlations with migraine characteristics in the M-ieNSAIDs group. The MLR and SVM models discriminated clinical efficacy of NSAIDs with an area under the curve (AUC) of 0.891 and 0.896, sensitivity of 0.971 and 0.833, and specificity of 0.629 and 0.875, respectively. These findings suggest that the efficacy of NSAIDs in migraine could be predicted using ML algorithm. Furthermore, this study highlights the role of amygdala-related neural function in revealing underlying migraine-related neuroimaging mechanisms.

## Introduction

Migraine is the leading cause of disability among young women. Moreover, it is the second leading cause of disability worldwide affecting over 15% of the population (GBD 2015 Disease and Injury Incidence and Prevalence Collaborators, 2016). It is a common prevalent neurological disorder characterized by recurrent moderate to severe headaches and is aggravated by physical activity. Non-steroidal anti-inflammatory drugs (NSAIDs), sedatives and opioids, are often used for the management of migraine (Tfelt-Hansen, 2021). In particular, NSAIDs are considered first-line drugs for the treatment of migraine attacks (Ashina, 2020). Nonetheless, NSAIDs have unsatisfactory therapeutic outcomes in a considerable percentage of the patients due to inter-individual variability (Biglione et al., 2020). Moreover, it has been shown that migraineurs who tend to overuse NSAIDs suffer from treatment-resistant headaches and cognitive decline more frequently (Cai et al., 2019). In addition, it was shown in an observational study that migraine patients with or without medication overuse had an increased risk of developing depression. Moreover, migraineurs with medication-overuse had a higher prevalence of depression (Mose et al., 2018). Of note, NSAIDs-induced addiction, cognitive dysfunction and psychiatric disorders are suggestive of central nervous system (CNS) abnormalities in migraine progression, which may lead to unsatisfactory results with the migraine-specific treatments. Furthermore, there is a need to have further understanding of inter-individual variability affecting the response to NSAIDs to allow optimization of therapy.

Endogenous bioactive lipids are critical mediators in hyperalgesia (Patel and Dickenson, 2021). The inhibition of the cyclooxygenase (COX) enzymes and subsequently the synthesis of prostaglandins are the mechanism of action of NSAIDs to tackle the most common forms of hyperalgesia. The COX metabolic hubs are involved in the ascending and descending pathways that modulate nociceptive signaling, including the paraventricular nucleus, prefrontal cortex, amygdala, thalamus, and periaqueductal gray (Buisseret et al., 2019). Due to the complex pathophysiology of migraine, it is described as a disabling neurolimbic pain disorder (Tolner et al., 2019). The amygdala, a core region of the neurolimbic system, plays a crucial role in regulating the top-down nociceptive pathway (Maizels et al., 2012; Minen et al., 2016). On the one hand, seed-based whole-brain functional connectivity (FC) and Granger causality analyses revealed that migraine sufferers exhibit aberrant connectivity between the amygdala and the higher cortex (Wei et al., 2020b; Huang et al., 2021). On the other hand, some studies suggested recurrent nociceptive input could affect the anatomical pattern of the amygdala, and these changes may explain the functional abnormalities in migraine patients (Jia and Yu, 2017). Besides, the amygdala is implicated in analgesic response to NSAIDs. Furthermore, the analgesic effects of NSAIDs are mediated *via* the descending pain inhibitory pathways (Hodkinson et al., 2015). Studies have shown that antinociceptive tolerance to NSAIDs is associated with disrupted amygdala-related FC patterns. However, studies on amygdala-related functional changes for predicting efficacy to NSAIDs in migraine have not been conducted. Multivariable analysis was used to detect group differences. However, neuroimaging-based predictions are required to identify differences at the individual level. Machine learning (ML) provides a complementary strategy to guide individual-level prediction by integrating neuroimaging and clinical features. Although several recent studies used ML methods to investigate migraine-related features, such as classification, frequency, and the efficacy of acupuncture (Chong et al., 2017; Mu et al., 2020; Yin et al., 2020), only a few studies used ML algorithms to predict the efficacy of NSAIDs in migraine.

In this study, the bilateral amygdalae were selected as the seed regions for FC analysis. The study only included patients with migraine without aura (MwoA), the most common subtype, to reduce potential interference due to heterogeneity. We hypothesized that the amygdala-related abnormalities could guide in predicting the efficacy of NSAIDs in MwoA patients. Herein, we established a conventional multivariable logistic regression (MLR) and support vector machine (SVM) models, to predict the efficacy of NSAIDs. These models could help clinicians optimize therapeutic effectiveness of medications used to manage migraine at the individual level.

## Materials and Methods

### Participants

A total of 73 patients diagnosed with episodic MwoA were recruited. A diagnosis of migraine was based on the International Classification of Headache Disorders, 3rd edition (ICHD-3) (Headache Classification Committee of the International Headache Society (IHS), 2013). To reduce potential physiological and pharmacological effects, the inclusion criteria were (1) MwoA patients not taking medications for at least 1 month before enrollment; (2) migraineurs who were headache-free for at least 3 days before and after the scanning. In addition, 33 healthy subjects (all right-handed) matched for age, sex, and education level were recruited as healthy controls (HCs). The exclusion criteria were: (1) comorbidities with other forms of headache; (2) history of psychiatric diseases or severe brain injury; (3) history of drug use disorders; (4) pregnant women, and (5) any contraindication to magnetic resonance imaging (MRI) examination. Each patient was required to keep a headache diary and complete a semi-structured questionnaire on demographic variables, clinical data, headache profile and past medical history. The study protocol was reviewed and approved by the ethics committee and review boards of the Affiliated Jiangning Hospital of Nanjing Medicine University. All study participants signed a written informed consent.

### Clinical Characteristics

All migraineurs underwent cognitive impairment screening using Montreal Cognitive Assessment (MoCA) to evaluate the cognitive condition. All the participants had MoCA scores of above twenty-five. In addition, the severity and impact of headaches were assessed using the Visual Analogue Scale (VAS) and the Headache Impact Test six-item scale (HIT-6). Furthermore, headache-related disability was quantified using the migraine disability assessment (MIDAS). The MwoA patients were instructed to record pain intensity before and 2 h after taking the medicine. Complete, partial, minimal, and no responses were classified as >75, 50–75, 25–50, and <25% reduction in pain intensity, respectively. Response to NSAIDs was defined as a 50% or greater reduction in VAS scores between the pre-treatment level and 2 h after taking medication.

### Imaging Data Acquisition

Imaging data were obtained using a 3.0 T MRI scanner (Ingenia) with an eight-channel head coil. The foam padding and earplugs were used to reduce head motion and scanner noise. Structural images were acquired with a three-dimensional turbo fast echo T1-weighted imaging sequence as follows: repetition time (TR)/echo time (TE) = 8.1/3.7 ms; slices = 170; thickness = 1 mm; flip angle (FA) = 8°; acquisition matrix = 256 × 256; field of view (FOV) = 256 mm × 256 mm. Functional images were obtained axially as follows: TR = 2000 ms; TE = 30 ms; slices = 36; thickness = 4 mm; FOV = 240 mm × 240 mm; acquisition matrix = 64 × 64; and FA = 90°. All participants were required to stay awake without thinking anything.

### Data Analyses

Data analyses were preprocessed using the resting-state functional MRI (Rs-fMRI) Data Analysis Toolkit plus (RESTplus^1^). The first 10 volumes were discarded to minimize the effect of signal instability. Slice-timing with the middle slice as reference and realignment for head motion correction were performed. Participants with head motion > 2.0 mm translation or a 2.0^°^ rotation in any direction were excluded. Then, the data were spatial normalized to the Montreal Neurological Institute template (resampling voxel size = ^°^3 mm^3^ × 3 mm^3^ × 3 mm^3^). Six head motion parameters and mean time series of white matter (WM) and cerebrospinal fluid (CSF) were included in the regression analysis to eliminate possible effects on the results. Subsequently, detrended, filtered (0.01–0.08 Hz), and smoothed with an isotropic Gaussian kernel [full width at half maximum (FWHM) = 6 mm] were implemented. The WFU_PickAtlas software was used to extract the seed regions of interest (ROIs) of the bilateral amygdalae. The mean time series of each amygdala was computed as a reference time course. After that, Pearson correlation coefficients were calculated between the mean signal change of each ROI and the voxels. Finally, Fisher’s z-transformation was applied to improve the normality of the correlation coefficients.

### Structural Analysis

Structural images were processed based on the voxel-based morphometry (VBM8) toolbox.^2^ Briefly, cerebral tissues were segmented into gray matter (GM), WM, and CSF using the unified segmentation model. GM and WM volumes were calculated by estimating these segments. The brain parenchyma volume was defined as the sum of GM and WM. Subsequently, the GM and WM images were smoothed using a 10-mm FWHM Gaussian kernel. Statistical analysis of the whole-brain GM was performed at the voxel level with family-wise error (FWE) correction for multiple comparisons (*p* < 0.05) or an uncorrected threshold (*p* < 0.001, cluster size > 100), with age, sex, and education as covariates of no interest.

### Construction of the Multivariable Logistic Regression and Support Vector Machine Models

Univariable regression analysis was performed using the demographics, clinical characteristics and abnormal FC patterns. Only variables with *p* < 0.05 in the univariate analysis were included in the MLR model to identify independent risk factors affecting NSAIDs efficacy. Additionally, the migraineurs were randomly assigned to the training group (80%) and testing group (20%). The SVM model was constructed based on the demographic variables, clinical features and abnormal amygdala-related FC patterns between the two migraine subgroups through a five-fold cross-validation strategy on the training group. Further, the training group was randomly divided into five equal sized subgroups. Four of these subgroups were used as an intra-training set, whereas, the remaining subgroup was used as an intra-validation set. In each run, four subgroups were used to develop the classifier while the remaining subgroup was used to verify accuracy of the classifier. Subsequently, the SVM model was tested using the testing group. Finally, the receiver operating characteristic (ROC) curves and values of sensitivity, specificity, and area under the curve (AUC) values were obtained.

### Statistical Analysis

The SPSS software (version 25.0) was used for statistical analyses. Differences in the demographic and clinical characteristics were analyzed using a one-way analysis of variance (ANOVA) or a two-sample *t*-test for normally distributed data. In addition, the Kruskal-Wallis test or Mann-Whitney *U*-test were used to determine differences in non-normally distributed data. The Chi-square test was used to determine differences in categorical variables. Significant voxel time courses (FWE correction for multiple comparisons, *p* < 0.05) were considered to determine the functional connection of the amygdala-related FC patterns. Age, sex, and education were considered as nuisance covariate. Furthermore, the Pearson correlation was used to analyze the correlation between the amygdala-related dysfunctional connectivity across the three groups and the corresponding clinical characteristics of each migraine subgroups. A *p*-value of less than 0.05 was considered statistically significant.

## Results

### Demographics and Clinical Characteristics

Three patients were excluded from the study due to excessive head movement. Therefore, the final cohort included 35 MwoA patients who were effectively managed with NSAIDs (M-eNSAIDs), 35 MwoA patients with ineffective response to NSAIDs (M-ieNSAIDs), and 33 healthy participants. The demographics and clinical characteristics of the study participants are shown in Table 1. There were no significant differences in demographic and clinical characteristics observed across the groups (*p* > 0.05).

**TABLE 1:** The clinical characteristics of the subjects.

	M-eNSAIDs	M-ieNSAIDs	HCs	*F*/*t*/χ2	*P*-value
Age (years)	33.91 ± 10.75	35.69 ± 11.47	36.30 ± 10.38	0.445	0.642
Sex (male/female)	3/32	4/31	2/31	0.669	0.907
Education (years)	13.54 ± 2.81	13.11 ± 3.08	12.39 ± 4.06	1.020	0.364
Disease duration (years)	7.94 ± 6.15	11.60 ± 10.08	/	−1.832	0.071
Frequency (times/month)	4.77 ± 3.15	5.23 ± 5.76	/	−0.412	0.682
Attack duration (hours)	17.31 ± 13.81	17.03 ± 15.05	/	0.083	0.934
VAS score	6.40 ± 1.54	5.97 ± 1.65	/	1.123	0.265
MIDAS score	38.97 ± 32.26	37.29 ± 31.78	/	0.220	0.826
HIT-6 score	57.57 ± 8.86	60.34 ± 9.41	/	−1.269	0.209
Gray matter (mm^3^)	613.88 ± 57.32	605.51 ± 47.31	595.21 ± 47.08	1.147	0.322
White matter (mm^3^)	516.05 ± 42.63	500.82 ± 54.28	502.22 ± 45.26	1.083	0.343
Parenchyma (mm^3^)	1129.93 ± 87.57	1106.33 ± 91.70	1097.43 ± 82.43	1.267	0.286

*HCs, healthy controls; HIT, headache impact test; M-eNSAIDs, migraine without aura with effective NSAIDs; M-ieNSAIDs, migraine without aura with ineffective NSAIDs; MIDAS, migraine disability assessment scale; NSAIDs, non-steroidal anti-inflammatory drugs; VAS, visual analogue scale.*

### Voxel Based Morphometry Results

There were no significant differences in total brain volume, including the GM volume, WM volume, and parenchyma volume among the three groups (Table 1). Moreover, structural analysis of the GM did not reveal any significant brain region, at a liberal threshold of *p* < 0.001 (uncorrected) or a stringent level of *p* < 0.05 (FWE correction).

### Functional Connectivity Between Effective Response to Non-steroidal Anti-inflammatory Drugs and Ineffective Response to Non-steroidal Anti-inflammatory Drugs

**FIGURE 1:**
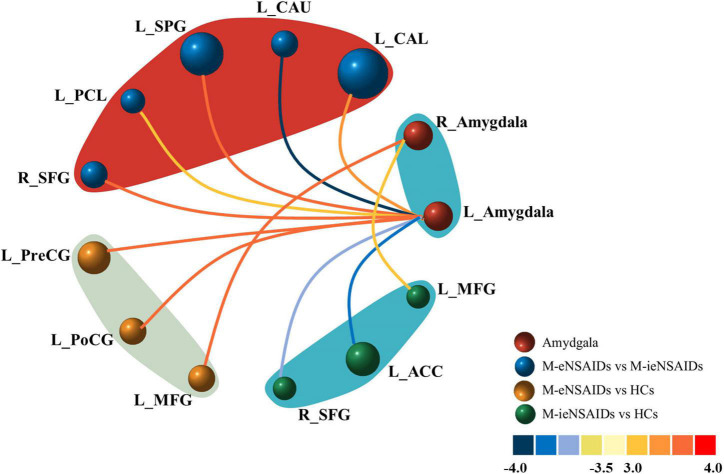
Abnormal functional connectivity of the left amygdala among the three groups. ACC, anterior cingulate cortex; CAL, calcarine sulcus; CAU, caudate nucleus; FC, functional connectivity; HCs, healthy controls; M-eNSAIDs, migraine without aura with effective NSAIDs; M-ieNSAIDs, migraine without aura with ineffective NSAIDs; MFG, middle frontal gyrus; NSAIDs, non-steroidal anti-inflammatory drugs; PCL, paracentral lobule; PoCG, post-central gyrus; PreCG, precentral gyrus; SFG, superior frontal gyrus; SPG, superior parietal gyrus; L, left; R, right.

The brain regions with increased FC of the left amygdala mainly located in the right superior frontal gyrus (SFG), left calcarine sulcus (CAL), left superior parietal gyrus (SPG) and paracentral lobule (PCL), and these with decreased FC located in the ipsilateral caudate nucleus (CAU) in the M-eNSAIDs patients compared to the M-ieNSAIDs patients. However, the right amygdala did not observe any disruption in FC between the two migraine subgroups (Figure 1 and Table 2).

**TABLE 2:** The regions with changed functional connectivity of amygdala among the three groups.

	Brain regions	MNI coordinates *x*, *y*, *z*	Cluster size	*T* value
**L_Amygdala**				
M-eNSAIDs vs. M-ieNSAIDs	L_CAL	−6, −96, 12	43	3.753
	L_CAU	−6, 15, 9	23	−3.924
	L_SPG	−18, −60, 66	37	3.648
	L_PCL	−6, −36, 72	21	3.242
	R_SFG	21, 42, −18	23	3.712
M-eNSAIDs vs. HCs	L_PreCG	−30, −18, 51	28	3.801
	L_PoCG	−27, −30, 72	23	3.849
M-ieNSAIDs vs. HCs	R_SFG	21, 42, −15	23	−3.647
	L_ACC	−6, 42, 3	20	−3.759
**R_Amygdala**				
M-eNSAIDs vs. HCs	L_MFG	−54, 30, 27	29	3.525
M-ieNSAIDs vs. HCs	L_MFG	−42, 48, −3	20	3.238

*ACC, anterior cingulate cortex; CAL, calcarine sulcus; CAU, caudate nucleus; FC, functional connectivity; HCs, healthy controls; M-eNSAIDs, migraine without aura with effective NSAIDs; M-ieNSAIDs, migraine without aura with ineffective NSAIDs; MFG, middle frontal gyrus; NSAIDs, non-steroidal anti-inflammatory drugs; PCL, paracentral lobule; PoCG, post-central gyrus; PreCG, pre-central gyrus; SFG, superior frontal gyrus; SPG, superior parietal gyrus; L, left; R, right.*

### Functional Connectivity Between Effective Response to Non-steroidal Anti-inflammatory Drugs and Healthy Controls

The brain regions with increased FC of the left amygdala primarily included the left precentral gyrus (PreCG) and post-central gyrus (PoCG), and with increased FC of the right amygdala included the left middle frontal gyrus (MFG) in the M-eNSAIDs patents, compared to the HCs (Figure 1 and Table 2).

### Functional Connectivity Between Ineffective Response to Non-steroidal Anti-inflammatory Drugs and Healthy Controls

The study found decreased FC of the left amygdala with the right SFG and left anterior cingulate cortex (ACC), as well as increased FC of the right amygdala with the left MFG in the M-ieNSAIDs patients, compared to the HCs (Figure 1 and Table 2).

### Functional Connectivity Between Migraine Without Aura Patients and Healthy Controls

The study found increased FC of the left amygdala with the right middle occipital gyrus (MOG) (*x* = 36, *y* = −96, *z* = 6; cluster size = 77; *t* = 4.3239), as well as increased FC of the right amygdala with the right MOG (*x* = 39, *y* = −93, *z* = 6; cluster size = 60; *t* = 3.7749), left IFG (*x* = −48, *y* = 45, *z* = 0; cluster size = 71; *t* = 3.4334) and left MFG (*x* = −51, *y* = 30, *z* = 33; cluster size = 61; *t* = 3.9930) in the MwoA group, compared to the HCs.

### Correlation Analysis

**FIGURE 2:**
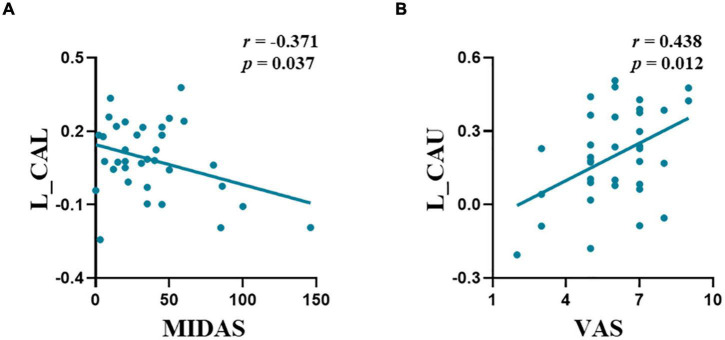
The abnormal functional connectivity patterns of the left amygdala showed significant correlations with migraine characteristics in M-ieNSAIDs **(A,B)**. CAL, calcarine sulcus; CAU, caudate nucleus; M-eNSAIDs, migraine without aura with effective NSAIDs; M-ieNSAIDs, migraine without aura with ineffective NSAIDs; MIDAS, migraine disability assessment scale; NSAIDs, non-steroidal anti-inflammatory drugs; PoCG, post-central gyrus; PreCG, pre-central gyrus; VAS, visual analogue scale; L, left.

Significant correlations were observed between the FC of the left amygdala-CAL and MIDAS scores (*r* = −0.371, *p* = 0.037) and FC of the amygdala-CAU and VAS scores (*r* = 0.438, *p* = 0.012) in the M-ieNSAIDs group (Figure 2). In contrast, there was no significant correlation between the FC of the right amygdala and the clinical characteristics.

### Multivariable Logistic Regression and Support Vector Machine Models

**FIGURE 3:**
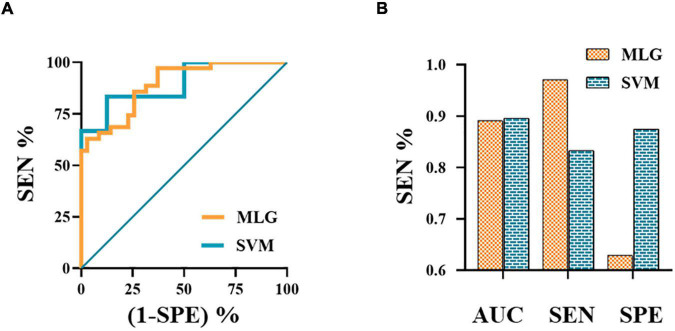
The ROC curves of MLG and SVM models **(A)**. The ROC curve of MLG model based on the anxiety scores and abnormal functional connectivity of the left amygdala with ipsilateral calcarine sulcus and caudate nucleus. The values of AUC, sensitivity and specificity of MLG and SVM were 0.891, 0.971, 0.629 and 0.896, 0.833, 0.875, respectively **(B)**. AUC, area under the curve; MLG, multivariable logistic regression; ROC, receiver operating characteristic; SEN, sensitivity; SPE, specificity; SVM, support vector machine.

The MLR analysis showed that abnormal FC patterns in the left amygdala with ipsilateral CAL and CAU were independent risk factors affecting the efficacy of NSAIDs (Table 3). The MLR and SVM models discriminated clinical efficacy of NSAIDs with an area under the curve (AUC) of 0.891 and 0.896, sensitivity of 0.971 and 0.833, and specificity of 0.629 and 0.875, respectively (Figure 3).

**TABLE 3:** Univariable and multivariable logistic regression models.

	*B*	SE	Wald	Unadjusted		Adjusted	
				*P*	OR (95% CI)	*P*	OR (95% CI)
L_Amygdala–L_CAL	0.680	0.202	11.344	0.001	1.973 (1.329–2.930)	0.012	1.811 (1.139–2.880)
L_Amygdala–L_CAU	−0.656	0.180	13.314	0.000	0.519 (0.365–0.738)	0.002	0.521 (0.347–0.783)
L_Amygdala–R_SFG	0.425	0.136	9.848	0.002	1.530 (1.173–1.996)	/	/
L_Amygdala–L_PCL	0.500	0.159	9.840	0.002	1.649 (1.206–2.253)	/	/
L_Amygdala–L_SPG	0.585	0.176	11.070	0.001	1.796 (1.272–2.535)	/	/

*OR, odds ratio; SE, standard deviation. ACC, anterior cingulate cortex; CAL, calcarine sulcus; CAU, caudate nucleus; CI, confidence interval; PCL, paracentral lobule; SFG, superior frontal gyrus; SPG, superior parietal gyrus; L, left; R, right.*

## Discussion

To the best of our knowledge, this was the first study conducted to predict the clinical efficacy of NSAIDs in MwoA patients. The study evaluated abnormal amygdala-related FC patterns between M-eNSAIDs and M-ieNSAIDs groups using the SVM classifier. The study revealed that the SVM classifier demonstrated predictive ability with an accuracy of more than 89%, treating abnormal amygdala-related FC patterns and clinical characteristics as features of interest. The present study demonstrated that the amygdala showed stronger FCs with some higher cognitive regions, including the limbic system, visual cortex and prefrontal cortex in MwoA patients compared to the HCs, consistent with previous studies (Wei et al., 2020b; Huang et al., 2021). The higher cognitive regions are involved in the top-down pain control circuit. Therefore, a disrupted circuit may lead to enhanced pain sensitivity. The right lateralization of the amygdala plays a dominant role in the modulation of pain processing and negative emotions (Jiang et al., 2014). In animal models, increased activation of the right amygdala induced by the extracellular signal-regulated kinase triggers hypersensitivity (Carrasquillo and Gereau, 2008). Moreover, blocking the activation of the right amygdala significantly decreases mechanical hypersensitivity (Ji and Neugebauer, 2009). These results suggest that the lateralized activation of the right amygdala mediates the generation and maintenance of pain. In the present study, there was increased left lateralization of the amygdala-related FC in the M-eNSAIDs group compared to the M-ieNSAIDs group. These results suggest that the neural function of the left amygdala plays an important role in determining the effectiveness of NSAIDs. In addition, the findings provide novel insights into the underlying neurophysiological mechanisms in migraine.

Compared to the M-ieNSAIDs group, these findings indicated a significant increase of FC pattern between the left amygdala and ipsilateral CAL in the M-eNSAIDs group. During the interictal and ictal periods, MwoA patients could develop visual, auditory and olfactory hypersensitivity. Photophobia is the most prevalent symptom. Then hyperexcitability of the visual cortex is considered the neural basis of hypersensitivity to light (Mulleners et al., 2001). Generally, the CAL is considered the core region of the primary visual cortex and is critical in the trigeminovascular pathway, which is essential for the underlying neuromechanism of migraine. Considering the complex functional connection of the dorsal and ventral visual pathways, the CAL may integrate multi-sensory information. Several previous studies revealed functional changes in the visual cortex in migraine patients, which are thought to modulate nociceptive perception (Wei et al., 2019, 2021). In this study, increased amygdala-CAL FC had a negative correlation with the MIDAS scores in M-ieNSAIDs patients. In addition, the MLR model showed amygdala-visual FCs which were shown to positively affect the efficacy of NSAIDs. These findings suggest that amygdala-visual dysfunctional patterns could affect the response to NSAIDs in migraine.

Furthermore, decreased interaction between the left amygdala and ipsilateral striatum region mainly located in CAU was observed in the M-eNSAIDs group. The putamen together with CAU forms the dorsal striatum. Functional neuroimaging studies (Chong et al., 2019) showed that central nervous system mechanisms are critical in understanding the pathophysiology of migraine and indicated the addictive behavior in the brain reward system, including the periaqueductal gray, limbic system and striatum. As a critical region in the reward circuit, the CAU is involved in regulating the transmission of nociceptive inputs to the limbic system from the brainstem. Therefore, abnormal FC patterns in these areas could directly affect the processing of nociceptive information in the reward circuit. Moreover, a previous study employing arterial spin labeling (ASL) (Hodkinson et al., 2015) revealed that hyperactivation of the limbic and striatum regions is involved in the neuro-regulatory mechanism of post-operative analgesia with NSAIDs. Similarly, the current study showed that a dysfunctional amygdala-CAU was an independent risk factor for ineffective treatment response in migraineurs. Moreover, it showed a significant positive correlation with pain severity. This suggests that severe headaches could promote the complexity of migraine treatment and reduce efficacy to NSAIDs. Consequently, ineffective treatment outcomes could increase the risk of using higher drug doses, which may cause medication-overuse headaches. Excessive use of analgesics is associated with some adverse events, such as hepatotoxicity, nephrotoxicity or abnormal striatal function (Fumal et al., 2006). These phenomena influence each other and perpetuate a vicious cycle. In this study, the M-eNSAIDs group showed significantly enhanced FC between the left amygdala and SPG and PCL compared to the M-ieNSAIDs group. The brain regions are located in the trigeminovascular pathway and are involved in the modulation of pain (Russo et al., 2018). Furthermore, MwoA patients receiving acupuncture treatment demonstrated significantly lower FC patterns between the bilateral amygdalae and pain-related regions (Russo et al., 2018), suggesting that inhibition of functional activity of amygdala-related brain networks could reduce neural hypersensitivity leading to analgesia. Therefore, disruption of amygdala-related FC could be used to develop an effective prediction model for individualizing treatment in MwoA and considered as a potential indicator for developing therapeutic strategies and reduce the side effects.

This study indicated some disrupted brain regions primarily in the executive control network (ECN), salient network (SN), and sensorimotor network (SMN) between migraineurs and HCs. Functional studies have demonstrated coordinated activation of several brain areas in response to somatic and visceral stimuli, including the thalamus, anterior cingulate cortex (ACC), insular cortex, sensory cortices, prefrontal cortex (PFC), basal ganglia, and limbic system (Maizels et al., 2012). This network of brain regions involved in both sensory-discriminative and emotional-affective aspects of pain is termed the “neurolimbic pain matrix.” This study demonstrated increased amygdala-functional changes during the resting state with left SFG and MFG in migraineurs. Previous studies showed GABAergic monosynaptic projections from the amygdala to PFC, which could contribute to the affective-motivational aspect of neuropathic pain (Seno et al., 2018; Gadotti et al., 2019). Furthermore, the studies showed amygdala-PFC–periaqueductal gray (PAG)–spinal cord (SC) pathway critical for pain hypersensitivity (Huang et al., 2019). For example, optogenetic and pharmacological studies exhibited that functional activation of the amygdala to PFC inputs was related to loss of descending inhibitory modulation of pain signals (Zhang et al., 2015; Huang et al., 2019; Coppieters et al., 2021). Inhibitory deficit results provide evidence supporting that enhanced input from the amygdala to PFC was a well-received underlying mechanism for the generation and maintenance of neuropathic pain (Cheriyan and Sheets, 2020), consistent with the findings of this study.

The PFC is crucial for executive functioning and pain processing, based on the GABAegic pathway and its dysfunctional connections within the PFC-limbic-PAG circuit (Ong et al., 2019). In line with the susceptibility and negative feedback regulation to stress-related response *via* hypothalamic-pituitary-adrenal (HPA) axis, amygdala activation, and PFC deactivation potentially disrupt the descending pain-inhibitory pathway causing nociceptive experience (Ji and Neugebauer, 2011; Cheriyan and Sheets, 2018). Hodkinson et al. (2015) reported increased cerebral blood flow (CBF) in the midbrain, amygdala and hippocampus, and decreased CBF in the insula, primary sensory cortex and PFC following treatment with analgesics. These differences may be attributed to ongoing pain after surgery. Furthermore, decreased amygdala-related hyperconnectivity has been observed after pain rehabilitation treatment, specifically between the left amygdala and right SFG and between the right amygdala and left MFG after acupuncture (Simons et al., 2014; Luo et al., 2020). While the above mentioned studies showed that suppressing the input from the amygdala to PFC might modulate the top-down inhibitory circuit. In this study, the M-ieNSAIDs group showed significantly decreased amygdala-PFC and amygdala-ACC connectivity patterns than the M-eNSAIDs group and HCs. The decreased amygdala-related functional connections may lead to ineffective treatment with NSAIDs in M-ieNSAIDs patients, implying other neural pathways may underlie the nociceptive mechanism, including the PFC and ACC. Of note, PFC and ACC are part of the mesolimbic reward circuit modulated by a dopamine neurotransmitter (DosSantos et al., 2017). Thus, decreased connectivity of the left amygdala with PFC and ACC may depict impairment of the reward circuit, which could explain for the ineffective response to NSAIDs treatment.

This study also revealed significantly increased FC between the left amygdala and ipsilateral PreCG and PoCG in the M-eNSAIDs group. The SMN, including the PreCG and PoCG, plays a major role in pain perception and the integration of sensory information. Furthermore, abnormal connections between the amygdala and SMN may partly explain the hypersensitivity in migraineurs to endogenous changes and exogenous stimuli. Besides, previous studies showed that the dysfunction of SMN played an important role in encoding nociceptive information. Moreover, amygdala-SMN connectivity may predict pain-intensity modulation (Gandhi et al., 2020; Wei et al., 2020a). Furthermore, fMRI and positron emission tomography-computed tomography approaches (Qin et al., 2008; Yang et al., 2012) demonstrated that acupuncture was effective in reducing pain intensity by modulating the amygdala-associated brain networks and deactivating the metabolism of SMN. Additionally, cellular mechanisms with increased neuronal excitability in the primary somatosensory cortex have been shown to target these microcircuits *via* inhibiting the SMN activity (Okada et al., 2021), consistent with this study. Therefore, the increased connectivity between the amygdala and SMN in M-eMwoA patients may represent impairment of the inhibitory pathway. Furthermore, these findings may provide insights for developing potential therapeutic targets. Based on the findings of this study, we concluded that alteration of amygdala-related FC patterns with the higher-level sensory cortex and prefrontal cortex were important central pathological features for distinguishing MwoA patients likely to have effective response to NSAIDs from MwoA patients likely to have ineffective response to NSAIDs. These results may help in further elucidating the functional characteristics of migraine and predicting individualized response to NSAIDs.

However, several shortcomings in this study need to be revealed. First, this study was cross-sectional in design and therefore could not reflect the causal relationship between the efficacy of drugs and changes in brain function. In the future, longitudinal studies are needed to verify the causal mechanism. Second, only MwoA patients in the interictal period were included. Therefore, future studies should include patients with other migraine subtypes and in other phases. Third, hormone levels and concomitant psychiatric diseases could potentially exacerbate migraine, influence treatment response and complicate migraine treatment (Yurt and Kaplan, 2018; Radat, 2021). Therefore, future studies should investigate the influence of the hormone profile and psychiatric disorders. Moreover, this study had a small sample size, especially male participants. Therefore, future studies should aim for a large sample size to avoid overfitting. Furthermore, this study did not consider the different distinct effects of each substructure of the amygdala on function. As such, future studies should focus on a more accurate analysis of the anatomical substructures of the amygdala. Finally, the drug history was not comprehensive enough to capture details such as drug dosages or the types of drugs. Therefore, future studies should conduct a comprehensive drug history.

In conclusion, our findings reveal that some amygdala-related FC patterns potentially act as objectively measurable neuroimaging indicators in NSAIDs treatment of MwoA patients. Besides, a combination of the SVM classifier and amygdala-related FC features may represent a valuable strategy for predicting the efficacy of NSAIDs and improving clinical decision-making.

## Data Availability Statement

The raw data supporting the conclusions of this article will be made available by the authors, without undue reservation.

## Ethics Statement

The studies involving human participants were reviewed and approved by Affiliated Jiangning Hospital of Nanjing Medicine University. The patients/participants provided their written informed consent to participate in this study. Written informed consent was obtained from the individual(s) for the publication of any potentially identifiable images or data included in this article.

## Author Contributions

H-LW, JL, and HZ designed the study. J-JW, G-PZ, and XG acquired the data. H-LW, Y-CC, and Z-ZH performed the data analysis. Y-CC, Y-SY, and XY interpreted the results. H-LW and C-HX prepared the manuscript. All authors contributed to manuscript revision and approved the submitted version.

## Conflict of Interest

The authors declare that the research was conducted in the absence of any commercial or financial relationships that could be construed as a potential conflict of interest.

## Publisher’s Note

All claims expressed in this article are solely those of the authors and do not necessarily represent those of their affiliated organizations, or those of the publisher, the editors and the reviewers. Any product that may be evaluated in this article, or claim that may be made by its manufacturer, is not guaranteed or endorsed by the publisher.

## Footnotes

1
http://restfmri.net/forum/

2
http://dbm.neuro.uni-jena.de/vbm
